# Development prospects of curable osteoplastic materials in dentistry and maxillofacial surgery

**DOI:** 10.1016/j.heliyon.2020.e04686

**Published:** 2020-08-11

**Authors:** A.V. Vasilyev, V.S. Kuznetsova, T.B. Bukharova, T.E. Grigoriev, YuD. Zagoskin, M.V. Korolenkova, O.A. Zorina, S.N. Chvalun, D.V. Goldshtein, A.A. Kulakov

**Affiliations:** a Central Research Institute of Dental and Maxillofacial Surgery, Moscow, Russia; b Research Centre of Medical Genetics, Moscow, Russia; c NRC “Kurchatov Institute”, Moscow, Russia

**Keywords:** Оsteoplastic materials, Bone cement, Alginate, Chitosan, Fibroin, Hydrogel, Cellulose, Hyaluronic acid, Collagen, Gelatin, Biomedical engineering, Biopolymer, Regenerative medicine, Orthopedics, Trauma, Dental materials, Dentistry, Materials science

## Abstract

The article presents classification of the thermosetting materials for bone augmentation. The physical, mechanical, biological, and clinical properties of such materials are reviewed. There are two main types of curable osteoplastic materials: bone cements and hydrogels. Compared to hydrogels, bone cements have high strength features, but their biological properties are not ideal and must be improved. Hydrogels are biocompatible and closely mimic the extracellular matrix. They can be used as cytocompatible scaffolds for tissue engineering, as can protein- and nucleic acid–activated structures. Hydrogels may be impregnated with osteoinductors such as proteins and genetic vectors without conformational changes. However, the mechanical properties of hydrogels limit their use for load-bearing bone defects. Thus, improving the strength properties of hydrogels is one of the possible strategies to achieve the basis for an ideal osteoplastic material.

## Teaser

Оsteoplastic materials; Bone cement; Аlginate; Chitosan; Fibroin; Hydrogel; Cellulose; Hyaluronic acid; Collagen; Gelatin; Biomedical engineering; Biopolymer; Regenerative medicine; Orthopedics; Trauma; Dental materials, Dentistry; Materials science.

## 1 Introduction

More than 75 million people in the United States, Europe, and Japan suffer from osteoporosis [1]. Due to the high prevalence of this disease, the annual number of vertebral, hip, and forearm fractures in Europe is expected to increase by 23% by 2025 [2], and with the loss of jawbone volume in osteoporosis implant surgery, more than half of the patients require bone grafting for implant placement [3]. A large number of clinicians are interested in new materials for treating extensive bone defects. Autogenous bone is still considered the gold standard for hard-tissue augmentation because it does not contain xenogeneic proteins, there is no need for special purification, and it includes osteoinductors for promoting osteogenesis [4]. However, the use of autogenous bone is associated with certain limitations. Bone harvesting requires an additional surgical procedure, with possible donor-site morbidity (including pain, blood loss, hematoma, infection, and so on), and the graft volume is restricted by the limited volume of the donor area [5,6]. In addition, when using bone chips, barrier membranes are required to exclude the ingrowth of soft tissues, and titanium mesh must be used to support the predetermined shape for directed bone regeneration [7, 8, 9]. These disadvantages limit the use of autologous bone and determine the need for advanced bone graft substitute materials. Several activated osteoplastic materials have pronounced osteoinductive and osteogenic properties [10], but they are not always convenient to use because of scaffold-related drawbacks. Among the developed scaffolds for osteoplastic materials, the most promising are moldable and curable compositions able to retain the predetermined form. They provide the convenience of their use without barrier membranes and titanium meshes, and the physical and mechanical properties of some thermosetting materials are similar to those of human bone. Thus, the choice of biocompatible materials that can be the basis for activated osteoplastic materials is an important task.

## 2 Classification of thermosetting osteoplastic materials

The main types of curable osteoplastic materials are hydrogels and cements. Hydrogels are three-dimensional hydrophilic polymeric networks capable of absorbing large amounts of water or biological fluids [11]. Bone cements are two-component systems comprising polymer powder and a liquid monomer. They can be cured by free-radical polymerization (poly (methyl methacrylate) [PMMA] cement) or by precipitation of calcium and phosphorus compounds (calcium-phosphate cement, CPC) [12].

## 3 Bone cement

Bone cements are widely used as materials for endoprosthetic replacement, vertebroplasty, and cranioplasty. The two main types are CPC and PMMA cements [12], with CPC divided further into two major subgroups: apatite and brushite cements [13].

### 3.1 Poly(methyl methacrylate) cements

The era of PMMA bone cements begins from the patent by Degussa and Kulzer (1943). They described the mechanism of polymerization of methyl methacrylate (MMA) at room temperature with addition of a co-initiator, such as tertiary aromatic amines. In 1958, J. Charley used cold-cured PMMA for total hip arthroplasty. In the 1970s, the U.S. Food and Drug Administration (FDA) approved the bone cement for use in hip and knee prosthetic fixation [14]. PMMA is formed by mixing liquid MMA monomer and powdered MMA-styrene copolymer. PMMA forms bone cement by a free-radical polymerization mechanism.

### 3.2 Calcium phosphate cements

CPCs were invented in the 1980s. Like PMMA cements, they consist of a powder and a liquid part; they form a paste and set under physiological conditions [12]. The powder phase consists of one or a combination of several calcium salts [15,16].

### 3.3 Properties of bone cements

Bone cements were conventionally used for replacement of the defects subjected to high mechanical loads (Table 1). PMMA cements have better mechanical properties (such as compressive strength and tensile strength) and shorter setting time than CPCs do. However, PMMA cements are not bioresorbable; they produce a fibrotic response and require a second surgery for removal [17].

**Table 1:** Mechanical properties of cements and hydrogels.

	Material	Pore size (μm)	Elastic modulus (MPa)	Tensile strength (MPa)	Compressive strength (MPa)	Ref.
Bone	Cortical bone	0.01–50	97.01–964.43	30.08–163.7	96.35–161. 44	[162, 163, 164]
	Trabecular bone	300–600	6.9–199.5	0.6–16.3	0.22–10.4	[165, 166, 167, 168]
Bone cements	Poly (methyl methacrylate)	50–3000	2140–3100	13–59	64–103	[169, 170, 171, 172]
	Calcium phosphate	200–600	180–8000	1–10	2.48–174	[173, 174, 175, 176, 177]
Hydrogels	Collagen	1–2.84	0.0005–0.027	–	0.007–0.012	[178, 179, 180, 181]
	Gelatin	45–250	0.011–0.081	0.047–1.2	0.3–7.5	[182, 183, 184, 185]
	Alginate	0.005–500	0.0002–0.003	25.8–58.3	0.012–0.18	[83, 121, 186, 187, 188, 189]
	Chitosan	20–500	0.039–0.087	0.013–35.2	0.18–2.78	[79, 190, 191, 192, 193]
	Fibroin	3.5403–86.4	0.369–1.712	4–7	0.024–1.111	[74, 194, 195, 196, 197]
	Hyaluronic acid	10–215	0.0005–0.0018	–	0.001–0.146	[110,198, 199, 200, 201]
	Polylactide-co-glycolide	50–450	22.663–74.949	3.19–15.347	0.6–2.4	[202, 203, 204]

CPCs are characterized by high porosity and, in contrast to PMMA cements, form chemical bonds with the components of the bone matrix. They are more biocompatible than PMMA [18,19].

CPC biodegradation occurs through extracellular fluid dissolution and cell resorption processes. The cement matrix is replaced by newly forming bone tissue [20]. Apatite calcium phosphate cements, in contrast to brushite, are characterized by a long period of biodegradation. Within six months after implantation, the mean extent of resorption of apatite cement is 5%, whereas that of brushite is 60% [21].

### 3.4 Disadvantages of bone cements

PMMA cements are not resorbable and do not promote osteogenesis. The polymerization of such types of cements is an exothermic process. These cements have no adhesive properties, so they need an irregular bone surface for proper mechanical fixation.

PMMA cements may cause bone cement implantation syndrome, characterized by hypotension, hypoxia, cardiac arrhythmia, and cardiac arrest. The reason for bone cement implantation syndrome was considered to be the release of the toxic MMA monomer into the bloodstream. More recent studies have shown that monomer emboli formation is possible along with the cytotoxic effect during the cementation of the prosthesis. Besides this, the use of PMMA cements provokes an increase in blood histamine concentration, which is a risk factor for cardiovascular complications in elderly patients [22,23].

The disadvantages of CPCs include the low biodegradation rate, small pore size, and lower strength properties than PMMA cements. In addition, cement particle migration to surrounding tissues with blood flow is possible in cases of single-walled bone defects [24].

### 3.5 Modifiers of physical and mechanical properties

It is possible to increase the compressive and tensile strength of bone cements by adding apatite, wollastonite, hydroxyapatite, calcium phosphate, silicon oxide, titanium, aluminum, and zirconium solid particles, as well as acrylamide and acrylic acid [25, 26, 27]. Addition of 2.5% hydroxyapatite in PMMA increases its compressive strength from 222 to 254 MPa, and the elastic modulus from 29 to 36 MPa [28].

The setting times of cements depend on the molecular weight, particle size, production method, powder and liquid ratio, humidity, temperature of the medium in which the mixing takes place, and the presence of additives [29,30]. Using distilled water as a liquid for CPC increases the setting time to longer than 30 min. However, using phosphate solutions of sodium, potassium, or ammonium, decreases the setting time to 5 min [31].

The porosity of CPCs may be enhanced by the addition of substances that form air bubbles (sodium dodecyl sulfate), liquids that do not mix with cement (for example, vitamin E solution), or the use of soluble crystals, such as sodium chloride, mannitol, and sodium salts of carbonates and phosphates [32, 33, 34, 35, 36]. Addition of sodium bicarbonate and citric acid polymer to PMMA cement results in pore diameters of 50 μm to 3 mm.

The bone adhesion of PMMA cement may be improved by bone pretreatment or the use of additional binding material. Adherence increases 3–5 times when using liquid acrylic material [37]. Etching bone with 37% orthophosphoric acid decreases adhesion but allows demineralization of the surface and exposure of collagen fibers. Further use of hydrophilic (hydroxyethyl methacrylate, 4-methacryloyloxyethyl trimellitic anhydride, glyceryl methacrylate) and hydrophobic monomers (bisphenol glycidyl methacrylate, trimethylene glycol dimethacrylate, polyethylene glycol dimethacrylate) leads to hybrid-layer formation with collagen fibers that increase adhesion between cement and bone [38]. Another way to modify PMMA cement is to add methacryloxypropyltrimethoxysilane and calcium acetate. It forms hydroxyapatite at the phase boundary and enhances adhesion [39].

Integration of 1-dodecyl mercaptan (Acros Organics, USA) or ammonium nitrate (Acros Organics, USA) decreases the temperature of the exothermic curing reaction; 1-dodecyl mercaptan acts as an agent that stops polymer chain formation, lowering the temperature by 4–6 °C, and an endothermic reaction occurs when ammonium nitrate is added, which decreases the exothermic reaction temperature from 96.5 °C to 73.6 °C [40].

### 3.6 Modifiers of biological properties

The low biodegradation rate interferes with the CPC replacement of bone tissue. Integration of rapidly degradable gelatin, polylactide-сo-glycolide, or glucono-delta-lactone accelerates cement degradation. Two weeks after implantation of the cement with 10% glucono-delta-lactone, 32.8% of it was replaced by new bone [41].

CPCs may be impregnated with osteoinductive proteins to accelerate bone formation [42, 43, 44, 45]. Adding rhBMP-2 to the cement used as a maxillary sinus graft resulted in 33% more newly formed bone in eight weeks than in controls without rhBMP-2 [46]. Osteogenesis may be activated by using human embryonic and bone marrow stem cells as components of tissue-engineering construction based on CPCs [47,48]. Biocompatibility of PMMA cements may be improved by the addition of hydroxyapatite, mineralized collagen particles, and vitamin E derivatives. Incorporation of methacrylic monomers derived from vitamin E can decrease the temperature of the exothermic reaction by 56–73 °C to reduce the likelihood of bone damage [49,50]. Gelatin, collagen, and bioactive glass particles accelerate proliferation and differentiation of cells and improve the biocompatibility of calcium phosphate bone cements [51,52].

CPCs are inferior to acrylic bone cements in their mechanical properties, but they have better biological properties and may be replaced by new bone. Thus, the modification of the physical and mechanical properties of CPCs is a promising direction for research. The modified CPCs could become alternatives to PMMA bone cements.

## 4 Hydrogels

Hydrogels are water-swollen polymeric networks consisting of cross-linked hydrophilic polymers. A greater number of cross-links between polymers increases viscosity and mechanical strength.

According to the cross-linking mechanism, hydrogels may be divided into groups that are self-setting or that set in response to external stimuli such as temperature, changes in pH, electromagnetic radiation, UV light, or ultrasound [53, 54, 55]. In addition, hydrogels can be classified as chemical or physical. Chemical hydrogels are formed by covalent or ionic cross-linking. Hydrogen bonds and hydrophobic interactions in amphiphilic block or graft copolymers form physical hydrogels [53,56, 57, 58].

Based on the source/origin, hydrogels can be grouped into natural and synthetic classes. Naturally occurring materials include polypeptides, polysaccharides, or composites [59]. Synthetic polymers are divided into biodegradable and non-biodegradable. Biodegradable polymers include polylactide, polyglycolide, and their copolymers polylactide-co-glycolide, polycaprolactone, and polycyanoacrylate. Polyvinyl alcohol, polyethylene glycol, poly (hydroxyethyl methacrylate), and poly-N-isopropyl acrylamide are non-degradable synthetic polymers [60]. The most often used polymers for creating hydrogels that set are collagen, alginate, chitosan, gelatin, hyaluronic acid, cellulose, and fibroin, as well as polyethylene glycol.

### 4.1 Cross-linking mechanism of hydrogels

#### 4.1.1 Thermosensitive hydrogels

The common characteristic of temperature-sensitive polymer networks is the presence of the hydrophobic groups such as methyl, ethyl, and propyl groups and so on. Examples of such hydrogels are materials based on poly-N-isopropylacrylamide. Its critical solution temperature is close to body temperature. Cross-linking the polymers provokes transition of spiral components into globules, thereby reducing the volume of the hydrogel [61]. It is possible to modify the phase transition temperature of the composite by copolymerizing it with hydrophilic or hydrophobic monomers [62]. Poly-N-isopropylamide has an autonomous self-healing ability that is lost after swelling in water. However, the addition of acrylic acid makes the self-repair of the composite possible after swelling [63]. Poly-N-isopropylamide has poor mechanical properties and is mainly used as a scaffold for the delivery of stem cells for the regeneration of bone tissue [64,65].

#### 4.1.2 pH-sensitive hydrogels

Changing the pH value leads to changes in the water content of hydrogels and their swelling extent; pH-sensitive hydrogels contain monomers with weak acidic or weak basic side groups containing charges that are pH-dependent, and pH-sensitive polymers consist of pendant acidic or basic groups. They have been most frequently used as scaffolds for drug delivery systems [66]. In addition, they may be used for bone tissue regeneration. An example of such hydrogels is a block copolymer of poly-e-caprolactone-co-lactide-polyethylene glycol-poly-e-caprolactone-co-lactide with the addition of pH-sensitive oligomers of sulfamethazine [67]. Under physiological conditions (pH 7.4 and 37.8 °C), the block copolymer solution rapidly forms a stable gel, whereas it forms a sol at pH 8.0 and 37.8 °C that allows it to be easily injected into bone defects. In vitro studies showed no cytotoxic effect of the composite at a polymer concentration of 200 mg/ml. It is biocompatible in vivo and does not produce signs of severe inflammation within seven weeks after subcutaneous injection. Hydrolysis of polyester blocks causes the implant to decrease in size but does not affect its shape [67].

#### 4.1.3 Photo-curable hydrogels

The photo-curable hydrogel is polymerized from photoinitiators that are activated by ultraviolet (UV) light and blue light. This method allows for better control of the curing moment. However, prolonged ultraviolet light exposure can damage the osteoinductors infused in the material due to free-radical formation on the surface [68]. An example of a UV-curable hydrogel is a methacrylate gelatin composite with the addition of an Irgacure 2959 photoinitiator (Ciba Specialty Chemicals, Basel, Switzerland), showing a high degree of survival and active cell proliferation in vitro. In vivo experiments proved a positive effect of the material on bone healing in 10 mm parietal bone defects. The formation of approximately 7 mm^3^ and 15 mm^3^ of bone tissue was observed after four and eight weeks, respectively [69]. Another way to manufacture materials cured by ultraviolet radiation is the modification of composites by their methacrylate action [70]. Methacrylate-modified chitosan and lactide solution in the presence of fibrinogen form a hydrogel by free-radical reactions induced by UV radiation. The compressive modulus of the material during 300 s of polymerization is 31.7 ± 0.9 kPa, but long polymerization times result in hydrogel deformation with decreased flexibility and increased stiffness [71].

#### 4.1.4 Sonication-induced hydrogels

Ultrasonication changes the structure of certain hydrogels. For example, sonication causes fibroin structure as ß-sheet that allows creation of a hydrogel capable of rapid gelation. Due to the fast gelation rate, it is possible to add growth factors such as BMP-2 and VEGF to the material without significantly reducing their activity during cross-link formation [72,73]. Fibroin hydrogels may also be used for cell delivery [74].

### 4.2 Chemical hydrogels with different types of cross-links

#### 4.2.1 Covalent cross-links

Collagen, gelatin, and chitosan can form covalent cross-links with various cross-linking agents, including genipin [75, 76, 77, 78]. Thus, the surfaces of materials based on chitosan and genipin attach twice as many cells. In addition, the corresponding modulus of elasticity for the genipin materials is 2.3 GPa, which is twice the value of the material based on pure chitosan [79]. The pore size of chitosan hydrogel varies from 100 to 150 μm, and when added to the composite, genipin decreases it to 50–100 μm. The porosity of the chitosan-based materials ranges from 24.28% to 46.25% for the materials without genipin and with 2% genipin, respectively [77]. The addition of genipin allows for the improvement of the biocompatibility of the chitosan-based materials. It was noted that gelation time, morphology, and rheological properties of the gels vary with the concentration of genipin, pH value, and the influence of different salts. In addition to genipin, the role of the cross-linking agent may be performed by formalin and glutaraldehyde, which are used in histological techniques [80].

Studies of the materials based on chitosan gel and genipin showed that the addition of hydroxyapatite increased the possible loading of the chitosan hydrogels with BMP-2 from 28% to 65%. The hydrogels with hydroxyapatite are characterized by a macroporous structure with pore sizes from 150 to 200 μm. Due to formation of hydroxyapatite nanocrystals, the materials possess large surface areas and complex structures. That allows for an increase in surface adsorption and release of BMP-2, which boosts osteogenic differentiation of multipotent mesenchymal stromal cells in vitro [81].

#### 4.2.2 Ionic cross-links

An example of a gel formed due to ionic cross-links is a polymer-based on alginate and calcium phosphate. In the study by A. Cardoso et al. (2014) [82], calcium phosphate was used as a mineral phase and a calcium donor for the alginate serving as a matrix. Cross-links were formed at room temperature and physiological pH. When using the material on the model of orthotopic osteogenesis, high biocompatibility of the gel was noted. Nevertheless, it was not able to withstand high mechanical stresses. (The dynamic modulus of elasticity was 500 Pa) It was necessary to monitor the pH and release of calcium ions in the replaced defect region to prevent an inflammatory response. After six weeks in an in vivo experiment, the composite degraded partially and was replaced by the newly formed bone tissue, which was in intimate contact with the remaining material. Addition of alginate hydroxyapatite improved the mechanical properties of the composite (the storage modulus reached values of 3 MPa), stabilized the hydrogel network, and lowered weight reduction by reducing the gel time (146 s, compared to 303 s for alginate) and the degree of swelling (about 5% for the polymer and more than 30% for alginate) [83].

Another method to form hydrogels is ionic and covalent cross-linking in one gel. For example, chitosan forms ionic cross-links with glycerophosphate and covalent cross-links with genipin [84]. These hydrogels not only have thermal sensitivity, which is characteristic of materials with ionic cross-linking, but also show improved mechanical properties and chemical stability specific for materials with covalent cross-links. The gelation time of the sample with glycerophosphate was 519 s, and addition of 0.15% genipin lowered it to 113 s; the elastic modulus values for the materials were 3 Pa and 1030 Pa, respectively. In addition, with varying degrees of genipin load, there were changes in the materials’ structures and their degradation rates. In vitro studies showed high biocompatibility of hydrogels. Gels were rapidly formed in vivo, with no changes in the positions of the gels or volumes within a week [85].

### 4.3 Physical hydrogels

Hydrogen bonds between the components form a hydrogel based on alginate and gelatin with the addition of silica particles. In the study by Lewandowska-Lancucka et al. (2017), the material was cured by UV radiation [86]. The gelation reaction lasted for 90 min. The material was prone to swelling (1596–2205%). The dynamic modulus of elasticity values of the composite varied from 6.88 to 9.03 MPa. The composite was biocompatible, but its cytotoxicity was higher than that of the pure-gelatin gel. Addition of silica particles, on one hand, increases the values of the modulus of elasticity compared to the pure alginate gels and chitosan; on the other hand, this addition induces mineralization of the composite.

An example of a physical hydrogel based on amphiphilic block and graft copolymers is a hydrogel based on polylactide-co-glycolide and polyethylene glycol. According to Dhillon et al. (2011), the mixing of polylactide-co-glycolide with a plasticizer such as polyethylene glycol allows production of the temperature-sensitive material with a transition temperature of 37 °C [87]. The particles of polylactide-co-glycolide and polyethylene glycol are mixed with the carrier solution. At room temperature, the material is plastic enough to be formed into shapes that solidify at 37 °C. The formation of the scaffold begins with the particles becoming soft and sticking to each other upon reaching the transition temperature. At this stage, the hydrophilic component of polyethylene glycol (PEG) is being washed out of the particles. Reducing the content of polyethylene glycol increases the transition temperature, and the particles return to the solid state. In the study by Dhillon et al. (2011) [87], the maximum compressive modulus of the material was 2 MPa after 2 h at 37 °C, which corresponds to that for the trabecular bone, and Young's modulus value was 40 MPa. According to Rahman et al. (2014) [88], the material was biocompatible and supported during in vitro cell growth and proliferation. The in vivo experimentation was performed on the calvaria critical-size defect mouse model. When 1 mg of BMP-2 was added to the composite, an increase in the bone volume of 55% was noted. (It was 31% for the material without BMP-2 when compared to spontaneous bone healing controls.)

### 4.4 Polymers for hydrogel scaffolds

#### 4.4.1 Collagen

There are 28 types of collagen fibers. Among these, type I collagen is the most prevalent type found in the extracellular matrix (ECM), especially in tissues such as tendon and bone [89,90]. The advantages of collagen are biocompatibility, high porosity, hydrophilicity, and degradability [91,92]. Collagen-based materials may be used for cell delivery in tissue engineering. Their structures allow for inclusion of osteoinductors and nucleotides while maintaining their activity [93, 94, 95].

The drawbacks of collagen include high biodegradation rate, poor mechanical properties, and the possibility of an immune response [96,97]. In vitro, complete degradation of the collagen scaffold can occur in just 3 h when using collagenase [98]. The full in vivo degradation of the materials based on collagen and glycosaminoglycan occurs within eight weeks [99].

#### 4.4.2 Gelatin

Gelatin is a product of collagen denaturation. Like collagen, it has favorable properties such as biocompatibility, enzymatic degradation, and non-immunogenicity as well as positive effects on cell adhesion. In vivo experiments showed that gelatin induced no inflammatory reaction during the process of degradation [100, 101, 102]. The gelatin hydrogels can be used to deliver growth factors, cells, and oligonucleotides [103,104], but they exhibit a high degradation rate, weak mechanical strength, and low shape stability, which limits their use for high-load-bearing defects [105,106].

#### 4.4.3 Hyaluronic acid

Hyaluronic acid is a copolymer of D-glucuronic acid and N-acetyl-D-glucosamine. It acts as a signaling molecule by regulating migration and proliferation of various cell types. The products of its degradation stimulate angiogenesis [107,108]. High–molecular weight hyaluronic acid also has anti-inflammatory properties [109]. It is possible to incorporate cells, growth factors, and nucleic acid into hydrogels based on hyaluronic acid [110, 111, 112]. Among its disadvantages are poor mechanical properties and a high rate of biodegradation [113]. Gels based on hyaluronic acid have a low modulus of elasticity values (85–140 Pa) due to high water content [114]. Thus, these hydrogels are inconvenient for replacing load-bearing bone defects.

#### 4.4.4 Alginate

Alginate is a natural polysaccharide consisting of α-D-mannuronic acid and B-L-guluronic acid. Alginate is biocompatible and non-immunogenic [115]. It has been widely used in biomedical applications, such as wound dressing and dental impression materials [116,117]. Due to swelling and the ability to form viscous solutions, alginic acid is used as a disintegrant in medical preparations, which can increase the rate of drug absorption. Alginate-based formulations are used for the symptomatic treatment of heartburn and to relieve symptoms of the gastroesophageal reflux disease [118]. In tissue engineering, alginate is used as a carrier for cells, growth factors, and genes [119,120]. The primary disadvantage of alginate is the slow resorption rate that is difficult to predict. However, the modified alginate hydrogels undergo degradation [121].

#### 4.4.5 Chitosan

Chitosan is a linear polysaccharide composed of randomly distributed β-1,4-linked D-glucosamine and N-acetyl-D-glucosamine. It is usually obtained from chitin through chemical deacetylation under heterogeneous conditions [122, 123, 124]. Chitosan exhibits biocompatibility and is capable of degradation in vivo without the formation of toxic products [125]. It suppresses the growth of bacteria and fungi due to ionic interactions of charged groups of the chitosan polymer chain with the walls’ components, which leads to release of the intracellular components and cell death. Chitosan also acts as a chelating agent and binds the microelements necessary for fungal growth. It can penetrate cell walls and binds to DNA, preventing synthesis of mRNA, and thereby affects the production of proteins [126, 127, 128, 129, 130, 131]. The chitosan-based materials also exert a hemostatic effect due to plasma absorption, binding, and coagulation of erythrocytes as well as adhesion of platelets and their aggregation [125,132,133].

Special attention should be given to the anti-tumor effects of chitosan due to several mechanisms: stimulation of interleukin 1 and 2 production, resulting in T-lymphocyte proliferation; apoptosis of tumor cells; and arrest of the cell cycle [134, 135, 136, 137, 138].

Unmodified chitosan can be dissolved only in acidic solutions due to the presence of strong intermolecular hydrogen bonds, which limits its use as an injectable hydrogel. Composites based on chitosan and glycosaminoglycans or other bioactive proteins can create suitable conditions for penetration, growth, and development of cells [139]. Chitosan hydrogel is a suitable matrix for delivery of cells, proteins, and genes [93,140, 141, 142].

#### 4.4.6 Fibroin

Fibroin is the protein of silk fibers produced by a variety of insects, scorpions, and spiders. It performs structural roles in cocoon and web formation. This protein exhibits biocompatibility, high rigidity and strength, and a controlled rate of degradation. Biodegradation of fibroin occurs by proteolytic enzymes without causing an inflammatory reaction [72,73,143]. Pore size and mechanical properties of fibroin scaffolds can be controlled by changing the concentration of fibroin and the size of the porogen particles. Increases in protein concentration and gelation temperature augment its mechanical compressive strength and decrease pore size [144]. In addition, this material does not require additional stabilization by chemical cross-linking. Fibroin hydrogels are used to deliver growth factors and stem cells [145]. The disadvantage of fibroin matrices is their low biodegradation rate, but chemical modification yielding a biodegradable substitute solves the problem [146].

#### 4.4.7 Cellulose

Cellulose consists of chains of glucose linked by β-1,4-linkages. It is the most abundant natural polymer on Earth and an important structural component of the primary cell walls of green plants [147]. The advantages of cellulose include biocompatibility, high hydrophilicity, and ability to adhere to cells [148, 149, 150]. It can be used as a scaffold for cells and growth factors [151,152].

Cellulose, however, has a low rate of biodegradation (more than 60 weeks) due to the lack of specific enzymes (hydrolases) in the human body. It also has a high density of nanofibrils, which limits the colonization of cellulose-based scaffolds by cells [153], and it is impossible to form a bond between cellulose and bone under physiological conditions [154]. Chemical modification of cellulose by phosphorylation enables formation of calcium phosphate at the border between bone and material and ensures satisfactory adhesion.

#### 4.4.8 Polyethylene glycol

Polyethylene glycol is a nontoxic and water-soluble ethylene glycol polymer. The FDA approved gels based on polyethylene glycol for the regeneration of bone tissue [155]. Positive properties of polyethylene glycol include biocompatibility, non-immunogenicity, and resistance to protein adsorption [156,157]. The rate of biodegradation depends on the cross-linking agents and can vary from 10 h to 22 days [158]. The materials based on polyethylene glycol and polylactide-co-glycolide are used as matrices for tissue engineering and protein-activated and gene-activated structures [159, 160, 161]. The disadvantages of the gels based on polyethylene glycol include low mechanical strength that limits their use for load-bearing defects.

## 5 Commercial materials for bone tissue regeneration

Currently, there are numerous commercial materials for bone augmentation. Particularly interesting materials are the composites containing hydrogels or the other materials capable of curing, along with a filler such as demineralized bone matrix or hydroxyapatite. The forms of these materials are easy-to-use paste and putty. However, it should be noted that, despite their diversity, only a small number of materials have been clinically tested and approved for use in clinical practice. The most popular bases for such materials are collagen, gelatin, carboxymethyl cellulose, sodium hyaluronate, and reverse-phase medium. The data on such materials is summarized in Table 2.

**Table 2:** Representative commercial materials for bone tissue regeneration.

Collagen-based	Putty (Tecnoss, Italy)	Bone paste made of micronized pre-hydrated collagenated cortico-cancellous bone (granulometry less than 0.3 mm) and additional collagen gel (Tecnoss® Gel 0); 80% bone mix and 20% collagen gel. *Clinical indications*: filling of post-extractive sockets, self-contained peri-implant defects and all defects that present a self-contained cavity.
	MASTERGRAFT® Putty (Medtronic Sofamor Danek, USA)	Made from a combination of medical grade purified collagen and biphasic calcium phosphate ceramic. *Clinical indications*: filling and/or augmentationof dental oral/maxillofacial bony tissue, including periodontal/intrabone defects, alveolar ridge augmentation, dental extraction sites, sinus lifts, and cystic defects.
Sodium hyaluronate-based	DBX (Synthes, USA)	Bone graft substitute composed of demineralized bone matrix (DBM) from human donors in a sodium hyaluronate carrier. *Clinical indications*: filling bony voids or gaps of the skeletal system that are not intrinsic to the stability of the bony structure.
	PepGen P-15 FLOW (Ceramed, USA)	Inorganic bovine bone matrix (ABM) coupled with a synthetic cell-binding peptide P-15, suspended in a sodium hyaluronate carrier. *Clinical indications*: treatment of intrabony periodontal osseous defects due to moderate or severe periodontitis, augmentation of bony defects of the alveolar ridge, filling tooth extraction sites, or sinus elevation grafting.
Reverse phase medium-based	DynaGraft® II (SeaSpine, USA)	Combination of demineralized bone matrix with a bioresorbable, reverse phase medium carrier. *Clinical indications*: filling gaps or voids that are not intrinsic to the stability of the bony structure, using as bone graft extender (extremities, spine, pelvis).
	Puros® Demineralized Bone Matrix with Reverse Phase Medium (DBM with RPM) Putty (Zimmer Biomet Spine, Inc., USA)	Combination of human bone tissue that has been demineralized and cancellous bone (from the same donor) mixed with poloxamer reverse phase medium *Clinical indications*: using as an autograft extender (i.e. extremities, posterolateral spine and pelvis) and as a bone void filler (i.e., extremities and pelvis) for bony voids or gaps that are not intrinsic to the stability of the bony structure.
Gelatin-based	BioSET® IC DBM (RTI Surgical, Inc., USA)	Composed of demineralized bone from human donors and a highly purified porcine gelatin carrier; available with or without cortical cancellous chips. *Clinical indications*: use as bone void filler for spine, extremities and joints.
	RegenaVate DBM Fill (Zimmer Dental, USA)	RegenaVate DBM Fill contains human demineralized bone matrix in an inert porcine gelatin carrier. *Clinical indications*: filling dental intraosseous cavities, oral and cranio-/maxillofacial defects.
Carboxymethylcellulose - based	C-Graft Putty (Citagenix Inc., USA)	C-Graft Putty is a demineralized bone matrix in carboxymethylcellulose carrier. *Clinical indications*: extraction socket grafting for ridge preservation, sinus augmentations and bone remodeling for subsequent implant placement.
	ExFuse II Putty (HansBiomed Corp., Korea)	ExFuse is composed of demineralized bone matrix and cancellous bone in carboxymethylcellulose carrier. *Clinical indications*: filling of dental intraosseous cavities, oral and maxillofacial defects, including periodontal/intrabony defects, alveolar ridge augmentation, dental extraction sites, sinus lifts, and cystic defects.

## 6 Conclusion

Two types of curable osteoplastic material bases are currently in use: bone cements and hydrogels. Compared to hydrogels, bone cements have high strength characteristics, but their biodegradation profiles and biocompatibility need improvement. Hydrogels based on natural polymers such as collagen, gelatin, hyaluronic acid, chitosan, and fibroin can form structures similar to the extracellular matrix. They can be used as safe and fully bioresorbable scaffolds for bone-tissue engineering and carriers of proteins and gene-activated structures. However, the mechanical properties of hydrogels are inferior to those of cements and limit their use for load-bearing bone defects. Numerous studies describe the modification of hydrogel strength properties with different fillers and cross-linking agents. Inventions in the future will create the basis for the ideal osteoplastic material. Among the existing polymers used to produce biocompatible hydrogels, the most promising are chitosan and collagen. The unique properties of chitosan include its ability to inhibit the growth of bacteria, fungi, and tumor cells. However, commercial osteoplastic materials based on chitosan hydrogel have not been presented yet. Collagen can be considered the most "physiological" hydrogel. It has excellent biocompatibility, and its degradation products can be used for bone matrix synthesis. No commercial collagen hydrogel-based composites activated by growth factors have been presented. However, reported case studies in this area suggest their development. These collagen hydrogel-based composites will affect the concept of treating patients with bone defects in dentistry and orthopedics.

## Declarations

### Author contribution statement

All authors listed have significantly contributed to the development and the writing of this article.

### Funding statement

This work was supported by the 10.13039/501100006769Russian Science Foundation [grant number 16-15-00298-P].

### Competing interest statement

The authors declare no conflict of interest.

### Additional information

No additional information is available for this paper.
